# The prevention of disease relapse after allogeneic hematopoietic cell transplantation in acute myeloid leukemia

**DOI:** 10.3389/fonc.2022.1066285

**Published:** 2022-11-30

**Authors:** Enrico Maffini, Margherita Ursi, Francesco Barbato, Michele Dicataldo, Marcello Roberto, Elena Campanini, Elisa Dan, Francesco De Felice, Serena De Matteis, Gianluca Storci, Massimiliano Bonafè, Mario Arpinati, Francesca Bonifazi

**Affiliations:** ^1^ IRCCS Azienda Ospedaliero-Universitaria di Bologna, Bologna, Italy; ^2^ Department of Experimental, Diagnostic, and Specialty Medicine, University of Bologna, Bologna, Italy

**Keywords:** allogeneic hematopoietic cell transplantation (HCT), acute myeloid leukemia (AML), disease relapse, donor lymphocyte infusion, hypomethylating agents relapse prevention in AML receiving allogeneic hematopoietic cell transplantation

## Abstract

Disease relapse represents by far the most frequent cause of hematopoietic cell transplantation (HCT) failure. Patients with acute leukemia suffering relapse after HCT have limited conventional treatment options with little possibility of cure and represent, de facto, suitable candidates for the evaluation of novel cellular and biological-based therapies. Donor lymphocyte infusions (DLI) has been one of the first cellular therapies adopted to treat post HCT relapse of acute leukemia patients and still now, it is widely adopted in preemptive and prophylactic settings, with renewed interest for manipulated cellular products such as NK-DLI. The acquisition of novel biological insights into pathobiology of leukemia relapse are translating into the clinic, with novel combinations of target therapies and novel agents, helping delineate new therapeutical landscapes. Hypomethylating agents alone or in combination with novel drugs demonstrated their efficacy in pre-clinical models and controlled trials. FLT3 inhibitors represent an essential therapeutical instrument incorporated in post-transplant maintenance strategies. The Holy grail of allogeneic transplantation lies in the separation of graft-vs.-host disease from graft vs. tumor effects and after more than five decades, is still the most ambitious goal to reach and many ways to accomplish are on their way.

## Introduction

Allogeneic hematopoietic cell transplantation (HCT) is a potentially curative treatment for acute myeloid leukemia (AML). However, despite the reduction in toxic death rates due to better supportive care and less intense preparative regimens, the corresponding figures of overall (OS) and leukemia-free survival (LFS) have not changed consensually over time (1, 2). This is because disease relapse incidence (RI) after allogeneic HCT remained stable, representing de facto, the most common cause of HCT failure today (3). Relapse risk is higher in the first 12 months after HCT and the prognosis for those patients is generally poor (4). A recent retrospective analysis conducted by Colleagues from Japan, showed that among 1265 adult AML patients transplanted in complete remission (CR), RI was 29% during the first 6 months after HCT, at a median of 6.1 months, then declined to less than 5% at 3-years. For those suffering relapse of the hematologic disease, OS was of 19% at 2-years, with time from HCT to relapse identified as the most relevant risk factor for survival (5). A recent single center analysis on 148 AML and myelodysplastic (MDS) patients relapsed after allogeneic HCT, showed a median survival of only 6 months (6). Several risk factors accounted for higher relapse risk, depending both on disease biology, and on transplant characteristics (7–9).

From a biological perspective, the mechanisms underlining AML relapse are multifaced and mainly attributable to the loss of Graft *vs.* leukemia (GvL) effect, causing the evasion of tumor cells from allorecognition (10). Genomic loss of mismatched HLA haplotype, HLA downregulation, inhibition of allogeneic T cells response through overexpression of inhibitory receptors, perturbation of anti and pro-inflammatory cytokines productions are the best-known alterations at the molecular level (**Table 1**). HLA loss may contribute to leukemic escape after allogeneic HCT with a variable frequency, depending on donor type and timing of relapse: studies conducted on patients allografted with T-cell replete haploidentical transplants, showed that AML cells can escape immunologic control through loss of the mismatched HLA in almost one third of cases, although the potent lymphocytolytic action exerted by cyclophosphamide against potent donor alloreactive T-cells clones (11, 12). Only recently, HLA loss has been described also in the setting of T-cell depleted haploidentical HCT (13). Other than haploidentical setting, HLA-loss has been observed in 4-12% with unrelated donors and only sporadically with HLA-matched siblings (14). Quite surprisingly, there are no reports of HLA loss in the setting of cord blood transplantation. Transcriptional downregulation of HLA-class II may lead to decreased antigen recognition by T-cells (15, 16). Irreversible genetic HLA loss in HLA-matched unrelated donor recipients suggests that allorecognition depends also on minor-histocompatibility antigens (17). In fact, loss of class II HLA expression, coupled with overexpression of inhibitory checkpoints molecules represent viable immunologic escape from donor-derive T-cells AML recognition (18). Recent evidence shows a thigh association between epigenetics and tumor immune escape, identifying PRC2 as a key driver of HLA class II loss of expression, modulating chromatin accessibility (19). AML cells of post-HCT relapsed patients express PD-L1, CD80 and CD112. Recent evidence suggests that *ex vivo* PD-L1 inhibition may elicit T-cells proliferation and IFN-gamma production, contributing to the restoration of GvL effect against tumoral cells (20). Leukemic cells may also escape GVL effect by acquiring immunoregulatory properties or losing sensitivity to immunologic cell-killing. The increase of anti-inflammatory cytokines on one hand and the reduction of pro-inflammatory cytokines on the other, both play an important role in the complex mechanism of leukemia immunological escape after HCT. The production of anti-inflammatory cytokines, such as TGF-β tend to attenuate immune responses and make leukemic cells less immunogenic for T-cell mediated attack (21, 22). Proinflammatory cytokines such as IL-15 are, conversely, produced by healthy cells, and contribute to remove leukemic blast cells and also to generate memory stem T-cells from naïve T-cells, contributing to the immunologic counterresponse directed to tumor cells (23). Lastly, the acquisition of novel oncogenic mutations as well as loss of tumor-suppressant genes in leukemic cells may contribute to relapse after allogeneic HCT. The relapse after allogeneic HCT of patients with MDS has been linked to the expansion of disease subclones (24). The comparisons of mutational and chromosomal alterations between diagnosis and post-HCT relapses in leukemic patients revealed modifications in the genomic asset (25).

**Table 1:** Tumor Mechanisms of Immune Evasion after allogeneic HCT.

Mechanism	Frequency	Therapy
Immune checkpoint inhibitors		
Epigenetic upregulation of inhibitory molecules (i.e. PD1; CTLA4)	Not assessed	Pharmacological Immune checkpoint blockade
> Anti-inflammatory Cytokines		
TGF-β attenuate immune-mediate leukemic cell killing	Not assessed	TGF-β pharmacological inhibitors
IL-4 and IL-10 antagonize immune tumor cell killing	Not assessed	IL-4/10 blockade
< Pro- inflammatory Cytokines		
IL-15 contributes to the immunologic counterresponse vs. tumor cells	Not assessed	IL-15 superagonist complex
Oncogenes		
Oncogene-driven immune escape; JAK2, KRAS, FLT3	Not assessed	driving signaling pathway Inhibition
Impaired tumor cell recognition		
Loss of HLA I expression	30% in T-Cell Replete-Haplo; 4-12% with Unrelated donors	Second Transplant
Downregulation of HLA class II	Up to 30% overall	therapeutic strategies to resensitize AML cells to GvL

## Donor lymphocyte infusions

The use of DLI has been reported more than three decades ago for the treatment of patients with leukemia relapsing after HCT, based on the rationale that GvL effect reduces the risk of disease relapse following HCT (26–29). From a biologic standpoint, DLI effect on malignancies is thought to be mediated primarily through reversal of T-cell exhaustion, while increased tolerance and anergy are some of the mechanisms involved in DLI resistance (30). Responder’s patients’ blood is enriched in late-differentiated T cells before the cellular product infusion and in early-differentiated T-cells after DLI, suggesting that the expanding T-cell populations are probably deriving from pre-existing clones rather than from DLI (31). The analysis of blood samples from patients relapsing after HCT revealed features of T-cell exhaustion of both CD4+ and CD8+ T-cells, with high levels of PD-1, T-cell immunoglobulin and mucin-domain containing-3 (TIM3), Cytotoxic T-Lymphocyte Antigen 4 (CTLA4), Lymphocyte Activating gene-3 (LAG-3) (32). These cells have a net reduction of their proliferative capacity and cytokine production. The T-cell subset most involved from such a perturbation are the antigen-trained effector and central memory (33). Patients responsive to DLI exhibit a reversal from the exhaustion state, with downregulation of exhaustion markers and recovery from function disability. The analysis of CD8+ T-cells TCR repertoire of DLI recipients is characterized by a lower diversity, suggesting, in the context of effective GvL with low or absent graft-versus-host disease (GvHD), a lower reactivity towards minor histocompatibility antigens (34). Patients receiving DLI for relapse of the hematological disease after HCT, the expansion of CD8+ clones after the infusion is a viable tool able to predict durable remission (35).

DLI are conventionally infused in two different scenarios: in patients deemed to be at higher risk of disease relapse after HCT, such as those with unfavorable cytogenetics and/or molecular profile, active disease at the time of transplant, or those receiving T-cell depleted grafts, DLI is given for prophylaxis of relapse. On the other hand, the persistence of measurable residual disease (MRD), or the presence of mixed chimerism (MC) after HCT, they both represent indications for DLI infusion in a pre-emptive therapy setting (**Table 2**). DLI demonstrated to be very limited in the treatment of overt hematologic relapse, as several reports described (52). A dose-escalation protocol is generally the first choice, to mitigate the risk of GvHD without compromising GVL. The most appropriate starting dose and the subsequent escalation is upon physician choice, depending on both clinical and biological variables such as disease characteristics, conditioning regimen adopted, donor type and, probably the most relevant single factor, the time from HCT. Timing of DLI administration is generally not inferior to six months after HCT, because the very high-risk of inducing acute GvHD. A putative role for such a situation may be played by antigen presenting cells, that are gradually replaced from host to donor origin. A lower first dose seems appropriate when infusing DLI from haploidentical donors (53). DLI may not be administered because of early disease relapse, active GvHD or history of severe GvHD, marked cytopenia and severe active infections. The incidence and severity of post-DLI GvHD is critically dependent on some key points: time from HCT to DLI, degree of HLA disparity and the aliquot of lymphocyte infused with each DLI (54). Retrospective analysis and registry data indicate that DLI provide greater chances of treatment success when the tumor burden is low or only at the molecular level (55).

**Table 2:** DLI studies in allogeneic HCT for AML.

Study	Patients	Indication	Med.N	Median CD3+/kg	Response	GvHD	OS
Guillame (36)	30 - prospective	Prophylaxis	3	n.a.	2-y RI: 27.6%	Gr.I-III acute:31% - cGvHD 2-y: 53%	2-y: 65.5%
Dominietto (37)	80 - retrospective	Treatment	n.a.	n.a.	MRD^+^ RI:36% MRD- RI:16%	n.a.	3-y MRD^+^: 78% 3-y MRD^-^: 26%
Mo (38)	101 - retrospective	Pre-emptive	n.a.	3.5x10^7^	3-y RI: 39.5%	aGVHD: 8.9% cGVHD 3-y: 21.6%	3-y DFS: 51.7%
Schmidt (39)	75 - prospective	Prophylaxis	2	n.a.	1-y RI: 27%	Gr.II-IV aGvHD: 25% cGVHD: 45%	2-y OS: 42%
Jedlickova (40)	46 - retrospective	Prophylaxis	3	MSD: 1x10^6^ MUD: 5x10^5^	7-y RI: 22%	Gr.II-IV aGvHD: 11.7% cGVHD: 23.5%	7-y: 67%
Mohty (41)	24 -prospective	Prophylaxis	1	n.a.	2-y RI: 54.2%	Gr.II-IV aGvHD: 16% cGvHD: 37.5%	2-y: 38%
Gilman (42)	35 - prospective	Prophylaxis	n.a.	5x10^4^	2-y RI: 14%	Gr.II-IV aGvHD: 11% cGvHD: 16%	2-y: 69%
Cauchois (43)	36 - retrospective	Prophylaxis	1	5x10^5^	1-y RI: 16%	1-y cGvHD: 33%	1-y: 76%
Yang (44)	34 - retrospective	Prophylaxis	n.a.	3.8x10^7^	5-y RI: 20.5%	Gr.II-IV aGvHD: 17.6%	5-y: 67.8%
Sheth (45)	224 - retrospective	Pre-emptive	2	1x10^6^	2-y: 38%	Gr.III-IV aGvHD: 13% - cGvHD: 37%	5-y: 54%
Zhang (46)	28 - prospective	Prophylaxis	1	2x10^7^	3-y RI: 26.1%	aGVHD: 25.8% - cGVHD 3-y: 21.6%	3-y: 48.9%
Bug (47)	18 - prospective	Prophylaxis	2	ArmA: 0.2x10^6^ ArmB: 0.9x10^6^	2-y RI: 20%	cGvHD 2-y: 29%	2-y: 81%
Kalin (48)	110 - prospective	Prophylaxis	2	n.a.	2-y RI: 35%	Gr.II-IV aGvHD: 24% - cGvHD: 22%	2-y: 50%
Rettig (49)	42 - retrospective	Pre-emptive	6	2.3x10^6^	2-y RI: 21%	aGvHD: 29% cGvHD 14%	Median: 40.9 mo.
Kryshnamurthy (50)	62 - retrospective	Pre-emptive	n.a.	1.5x10^6^	n.a.	aGvHD 7.5% - cGvHD 29%	5-y: 83%
Yang (51)	250 - retrospective	155 pre-emptive 95 prophylaxis	n.a.	n.a.	3-y RI: 42.8%	n.a.	3-y: 50.8%

No., number; Med.N, median number; AZA, azacytidine; DEC, decitabine; PBS, panobinostat; RI, relapse incidence; cGvHD, chronic graft vs. host disease; aGvHD, acute graft vs. host disease; OS, overall survival; Gr, Grades.

### Pre-emptive DLI

Pre-emptive DLI has been used to eradicate MRD and promote donor chimerism after allogeneic HCT, with relevant benefits for recipients, respect to therapeutic DLI (56, 57). A recent study compared therapeutic DLI respect to pre-emptive strategy for the treatment of molecular and hematological relapses. The Authors reported on a single-center experience conducted on 342 adult AML patients allografted between 2009 and 2017. A total of 93 patients received DLI, of whom 42 as a pre-emptive infusion (based on molecular monitoring) and 51 for frank hematologic relapse. Median OS was 40.9 months *vs.* 10.4 months, for pre-emptive infusion and for frank relapse, respectively; of note, survival was inferior for those with molecular MRD respect to those deemed at high-risk from a clinical standpoint or with mixed chimerism only (49). Similarly, DLI was given to patients who showed early signs of relapse such as persisting MRD, reappearance of molecular markers of the leukemia, or mixed chimerism (58). A recent meta-analysis investigating the role of preemptive DLI for adult AML and MDS patients, including 222 patients from 8 studies, showed a survival of 72% after a median follow up of 46 months, with acute and chronic GvHD incidence of 20% and 25%, and non-relapse mortality (NRM) rates of 52% (59). A single-center retrospective analysis from China compared the outcomes of adult leukemia patients with high-risk features treated with preemptive compared to a prophylactic approach. One hundred and fifty-five patients received preemptive DLI *vs.* 95 patients receiving prophylactic DLI. The patients were stratified into five risk groups. There was an overall advantage for patients of very high-risk group receiving prophylactic DLI in terms of survival and relapse incidence. This strategy was also useful for MRD-positive patients before HCT (51). Colleagues from King’s College reported on the outcome of 113 adult AML/MDS patients treated with preemptive (n= 62) or therapeutic (n= 51) DLI after reduced intensity conditioning (RIC) HCT, with anti-thymocyte globulin (ATG) (n= 14) or Alemtuzumab (n= 99) for GvHD prophylaxis. Preemptive DLI were given to restore a mixed donor chimerism. Patients treated with preemptive DLI experienced OS of 80% and leukemia-free survival (LFS) of 65%, respect to a dismal 40% OS of those receiving therapeutic DLI, with a 5-year relapse rate of 69%. Cumulative incidence of GvHD for preemptive DLI was 31% (50). A retrospective analysis of preemptive DLI in 80 adult patients with acute leukemia after HCT demonstrated survival advantage for patients with positive MRD, receiving DLI. The MRD monitoring was performed on WT1 transcripts. The cumulative incidence of relapse was 16% in MRD-negative patients, compared with 6% of MRD-positive patients treated with DLI and 63% of MRD-positive patients without DLI (37). In order to maximize the eradication power of preemptive DLI respect to MRD, Investigators from China evaluated the combination of antiblastic chemotherapy and modified DLI in ATG-based T-cell replete allogeneic HCT in a comparative study between chemotherapy and DLI *vs*. DLI alone in high-risk AML/MDS, with similar results in terms of MRD-negativity (38).

### Prophylactic DLI

In the setting of very high-risk acute leukemia the infusion of DLI may be considered within a very strict time frame, very close to HCT. Patients harboring unfavorable cytogenetics, poor-risk mutations at the molecular level or diseases in florid expansion at the time of transplantation, need any effort to minimize disease relapse occurrence. Prophylactic DLI play a delicate role in such a complex scenario. Schimd et al. elaborated a sequential chemotherapy regimen followed by RIC HCT and ATG infusion (39). The early withdrawal of immunosuppression followed by prophylactic escalated dose DLI if no GvHD occurred was planned. A total of 75 AML patients with high-risk features were treated, twelve received the planned DLI. Two-year OS and LFS were 40%. Cumulative incidences of grade II–IV acute GvHD and chronic GvHD were, respectively, 49% and 45%. In a subsequent, recent retrospective analysis conducted on 45 high-risk AML and MDS patients, the majority with active disease at HCT, transplanted with the FLAMSA-RIC platform followed by DLI the Authors compared their outcome with a historical group of similar patients who have undergone the FLAMSA-RIC protocol without DLI. Long-term results were in favor of patients receiving DLI, with 7-year OS and LFS of, 78% *vs*. 34% and 68% *vs*. 38%, respectively (40). In an attempt to ameliorate the long-term clinical outcomes of FLAMSA-RIC platform and to reduce the total body irradiation (TBI) and amsacrine-associated toxic rates, The French published the results of a multicenter prospective phase 2 study, evaluating the efficacy of an alternative sequential regimen of clofarabine-based chemotherapy, with cytarabine and busulfan-based RIC allogeneic HCT, followed by prophylactic DLI for AML in primary induction failure. With a median follow-up of 24.6 months, the 2-year OS and LFS were 38% and 29%, respectively, with 2-year NRM of 12% (41). A Chinese study examined prophylactic use of decitabine followed by DLI was planned in 28 adult AML patients with TP53 or epigenetic mutations. No DLI-associated pancytopenia was observed. The cumulative incidences of grade II-IV and III-IV acute GvHD at 100 days post-DLI were 25.8% and 11%, respectively. Chronic GvHD incidence at 3 years was 21.6%. Non-relapse mortality and relapse at 3 years post-DLI were 25% and 26.1%, respectively. The 3-year LFS and OS were 48.9% and 48.2%, respectively. Acute GvHD (HR: 2.30, p = 0.016) and relapse (HR: 2.46, p = 0.003) after DLI were independently associated with inferior OS (46). Researchers from North Carolina reported on a phase I/II study of prophylactic DLI followed by methotrexate for GvHD prophylaxis after CD34-selected haploidentical donor transplant. A prophylactic DLI was given between day +30 and +42. Rituximab was given with DLI for the last 10 patients. The goal of the study was to determine a DLI dose that would result in a CD4+ cell count > 100/µL at Day +120 in ≥ 66% of patients with ≤ 33% grade II-III, ≤ 17% grade III, and no grade IV acute GvHD by Day +180. Thirty-five patients with malignant (n = 25) or nonmalignant disease (n = 10) were treated after CD34-selected haploidentical donor peripheral blood stem cell transplant. The DLI dose of 5 × 10^4^ /kg met the CD4/GvHD goal with 67% of patients having CD4+ cells > 100/µL and 11% grade II-IV acute GvHD. The cumulative incidence of chronic GvHD was 16%. Fatal viral and fungal infections occurred in 11%. The 2-year estimated overall survival was 69% and the relapse rate was 14% for patients in remission at transplant. There was no effect of NK alloreactivity on relapse (42). Jaiswal et al. evaluated the role of prophylactic DLI in PT-Cy-based T-cell replete HCT. The Authors reported on twenty-one AML patients receiving modified DLI, with ongoing immunosuppression with cyclosporine. Relapse incidence was 21% at 1-year, with acute GvHD incidence of 31%, with first DLI infused at day+21, suggesting that concomitant GvHD prophylaxis might control alloreactive T-cells causing GvHD (60). Cauchois er al. described a cohort of 36 patients affected by high-risk hematologic malignancies (AML, n= 21) allografted from haploidentical donors with PT-Cy based GvHD prophylaxis, receiving dose escalated DLI in a prophylactic setting. The one-year relapse incidence (RI) was 16%, while LFS was 76% (43). Researchers from China showed encouraging results from a matched-pair analysis of a cohort of 34 AML patients treated with prophylactic DLI after ATG-Fresenius based haploidentical HCT. Five-year OS and LFS were both superior for the experimental arm, with a significant lower rate of RI. Cumulative incidence of grades III-IV acute GvHD was 9.1%. In the multivariate analysis, DLI was an independent variable associated with higher LFS, OS and relapse (44). A recent retrospective comparative analysis demonstrated that the timely administration of prophylactic DLI for relapse prevention in mixed chimerism after unrelated donor HCT with alemtuzumab for GvHD prophylaxis, is associated with longer OS, LFS and lower RI respect to patients with MC who did not receive DLI, and superimposable to those achieving complete chimerism after HCT (45). EBMT recently published results from a retrospective analysis conducted on 173 patients transplanted from haploidentical donor with post-transplant cyclophosphamide as GvHD prophylaxis. Indication for DLI were prophylactic (34.3%), preemptive (11.6%), and therapeutic (54.1%). Active disease was the most important single factor associated with worse survival in all three settings. Acute GvHD rates were superimposable but a trend towards higher chronic GvHD was observed for those in the prophylactic group (61).

### Donor-derived NK DLI

The modification of the cellular product through cell selection has shown in the last few years promising anti-tumor effects with less attrition towards graft-versus- host reactions respect to conventional DLI (62). The rationale behind NK-DLI is the strong alloreactivity exerted by NK cells respect to tumor antigens, without inducing high rates of potentially fatal GvHD. The problem with such a therapeutic modality lies in the short-persistence of NK cells, mainly due to the inhibition played by regulatory T-cells (Treg) and myeloid suppressor cells on one side, and by tumor-induced NK cells anergy on the other (63–65). Several strategies have focused on generating large number of NK cells persisting *in vivo*, to retain their efficacy in the long-term. NCT018853358 was a phase I clinical trial evaluating the safety of donor-derived NK cells, ex vivo activated by IL-2, as maintenance therapy after allogeneic HCT. A total of 16 patients received DLI-NK between day +60 and +90 after HCT. Four patients developed chronic GvHD (including one severe). At last contact, 11 out of 16 patients were in CR without GvHD (66). Shapiro et al. showed excellent outcomes for patients affect by AML, infused with NK cells followed by IL2 administration to expand and maintain the transferred cell product, in the context of haploidentical HCT. The Authors incubated NK cells with IL-12, IL-15 and IL-18 to generate a cytokine-induced memory-like phenotype and treated so far six patients. No cases of GvHD developed but almost all patients experienced pancytopenia (67). To overcome the limited therapeutic potential of AML immunotherapy involving NK cells due to very low numbers of harvested cells in peripheral blood, Colleagues from MD Anderson employed a method to obtain high doses of NK cells, using K562 feeder cells expressing membrane-bound IL21 and 4-1BBL and presented their results of a phase I/II study and long-term follow-up. A total of 25 patients with myeloid malignancies receiving haploidentical HCT. Three doses of donor NK cells were administered on days −2, +7, and +28. After a median follow-up of 24 months, the 2-year RI was 4% vs. 38%, and LFS was 66% vs. 44% respect to contemporaneous 160 case-match controls from CIBMTR dataset. Acute GvHD occurred in ten patients (nine had grade 2 and one with grades 3/4). The patient who developed severe acute GvHD was a male with an older female donor. No patient developed chronic GvHD; these highly activated and cytotoxic “superbright” NK cells upregulated CD56 expression, CD16 and cell surface activating receptors (NKp30, NKp44, DNAM-1, NKG2D, and others). They were hyper functional compared with NK cells from PB, possessing high cytotoxicity and cytokine production (68).

Several ongoing clinical trials are evaluating the role of NK-DLI in terms of safety and efficacy against myeloid malignancies relapsing after allogeneic HCT. Investigators from Basel are performing a phase I/II clinical trial of the application of expanded NK cells following haploidentical HCT for AML/MDS (NCT03300492). The ELIT-AML01 trial (NCT03597321) is a French-based prospective multicenter randomized study examining the role of early prophylactic DLI after HCT for AML patients. Investigators from North America are examining the efficacy of NK-DLI administered with Toll-like Receptor 9 (TLR9) agonist from a 7-8/8 HLA-matched related/unrelated donor or 4-6/8 HLA-matched related donor, following RIC or nonmyeloablative HCT for myeloid and lymphoid diseases in a randomized phase II study (NCT02452697). A phase I study on manipulated DLI, is investigating if DLI depleted of TCR-αβ T cells and B cells can be infused on Day 28 following allogeneic HCT without inducing grade III-IV acute GvHD. The study will treat ten participants, five of which will have 10/10 HLA matched sibling donors while the others will have haploidentical donors (NCT03939585)

### Other manipulated DLI

In an attempt to reduce alloreactivity, the blockade of T-cells costimulatory molecules, such as cytotoxic T-lymphocyte-associated protein 4-immunoglobulin (CTLA4Ig), has been explored in pediatric haploidentical HCT, with acceptable results. Based on such a rationale, Colleagues from India published their experience of a comparison of two DLI-based clinical protocols, evaluating the efficacy of prophylactic early conventional DLI *vs.* CTLA4Ig-primed DLI. Supported by preclinical data, indicating a sparing-effect played by CTLA4Ig respect to NK cells, the Authors showed lower NRM (4% *vs.* 14.4%) and RI (15.7% *vs.* 31.1%) in CTLA4Ig-DLI group, as well as acute and chronic GvHD in the CTLA4Ig-DLI group (9.6% and 15.3% vs. 18.8% and 36.5% in the DLI group, respectively (69, 70). Investigators from Montreal developed a haploidentical, naïve T cells-enriched product, depleted of recipient-alloreactive T cells, called ATIR101 and infused it after a T-cell depleted haploidentical HCT in a Phase II multicenter study of 23 patients with acute leukemia, comparing clinical results with an observational cohort of HCT recipients. NRM at 1-year was inferior for patients receiving ATIR101 respect to recipients of conventional haploidentical HCT, as well as improved GRFS (71). A Phase I dose-escalating study explored safety and efficacy of naïve T-cells depleted DLI, following HLA-identical nonmyeloablative HCT for hematologic malignancies. One patient developed grade 2 acute GvHD of skin and GI, one moderate chronic GvHD of the lungs following the DLI. After a median follow-up of 2.8 years, 2-year progression-free and overall survival were 50% and 68.8% (72). Another phase I trial explored the combinatorial effects of the co-infusion of dendritic cells followed by DLI in a group of 16 patients with disease relapse after HCT. Of 14 evaluable patients, four achieved long-term remission, with one patient developing grade II acute GvHD (73). Citokine-induced killer (CIK) cells are another type of adaptive non-specific immunotherapy with potent anti-tumor activity against hematological malignancies (74). They are made up of cells bearing the CD3^+^CD56^+^ phenotype along with up-regulation of the NK cell receptor NKG2D. Their mechanism of action is not MHC-restricted, and possesses the dual capability of both T-cells and NK-cells (75). A comparative German-based study on the efficacy of CIK and DLI in hematological patients (AML, n= 31) with hematologic or molecular relapse after HCT, or active disease at HCT, showed superior survival rates, with 6-months OS of 77% and 57% in favor of CIK, with 81% vs. 50% for those patients receiving prophylactic infusions. A total of 19 DLI (35%) and 9 CIK cell recipients (25%) developed acute GvHD, with grades III-IV in 5 DLI recipients and one CIK cell recipient (76). CIK cells have also been tested in an Italian-based multicenter phase II clinical trial, where 74 relapsed patients after HCT were scheduled for receiving a sequential infusion of two DLI and three CIK cells. Forty-three patients received all planned infusions. Twelve patients developed acute GvHD, with five grades III-IV; eleven patients developed chronic GvHD. Disease control for this difficult to treat selected patient population was not completely satisfying, with 3-year OS and LFS of 40% and 29%, respectively for CIK cells (77). The use of CAR-T cells for AML treatment is still an unmet need, due to unique biologic leukemic cells characteristics, that make them an arduous target for biologic treatments. Car-engineered CIK cells with transposone technology are currently being explored in pre-clinical studies (78).

## Hypomethylating agents

### Hypomethylating agents

Nucleoside analogs of cytidine, hypomethylating agents (HMA) are DNA methylation blockers, approved for the treatment of AML and MDS. Their immunomodulatory properties make them appealing molecules in the HCT context, as pharmacologic agents employed in the maintenance phase after transplant. HMA are capable of up-regulate several key elements involved in immunogenicity and immune recognition, such as HLA class 1 antigens and tumor-associated antigens such as cancer-testis antigen (79, 80). Moreover, HMA can induce upregulation of both attractive chemokines such as C-X-C Motif Chemokine Ligand 9 (CXCL9) and immune checkpoints and respective ligands, such as Programmed cell death protein 1 (PD-1), and its respective ligand PD-L1 (81). Several reports indicate that HMA can also restore the expression of a group of NK ligands and killer Ig-like receptors, modulating, de facto, the innate immune system (82). Lastly, HMA have shown to induce both in animal models and in human patients, a regulatory phenotype on mature CD4+ T-cells and effector memory phenotype on CD8+ T cells (83, 84).

Colleagues from MD Anderson published more than one decade ago the first experience with HMA in the post HCT setting. The report included 17 patients allografted for AML and treated with subcutaneous azacytidine (AZA), both as salvage for relapsed disease (n= 9) or as maintenance treatment (n= 8). After a median follow-up of 16 months after HCT, one-year LFS and OS were 55% and 90%, respectively, without relevant adverse events, nor GvHD exacerbation observed (85). The same Group published the results of a phase I study involving 45 high-risk AML and MDS patients (67% with progressive disease) undergoing allogeneic HCT, receiving daily AZA starting from day +40 after HCT. The Authors concluded that AZA at 32 mg/m2 for 5 days, for at least 4 cycles could prolong survival estimates for such high-risk patients (86). Decitabine (DEC) role as maintenance agent after HCT has been investigated analogously several years ago in a phase I single-Center trial among 24 AML/MDS adult patients. After a median follow-up of 16.7 months, the rate of complete remission was 28%, and 2-year OS was 56%. Most toxicities appeared at maximum dose of 15 mg/m2 (87). The first RELAZA trial and the subsequent, RELAZA 2, investigated AZA as a maintenance agent after allogeneic HCT in patients with advanced MDS and AML, in a pre-emptive setting. RELAZA enrolled 37 adult AML patients receiving a RIC HCT between 2007 and 2010, and all of them had chimerism level measured on peripheral blood dropping below 80%. After a median of 4 cycles of AZA, 80% of patients had stable or improved MRD, without hematologic relapse. No new onset of GvHD was reported, among those without a previous manifestation of GvHD (88). In the subsequent RELAZA 2, a total of 60 AML and MDS MRD-positive patients in hematological CR did receive AZA 75 mg/m^2^ per day subcutaneously on days 1-7 of a 29-day cycle for 24 cycles. The primary endpoint was the proportion of patients who were relapse-free and alive 6 months after the start of pre-emptive treatment. With a median follow-up of 13 months after the start of MRD-guided treatment, LFS at 12 months was 46% in patients who were MRD-positive and received AZA. With a median time to relapse of 422days after HCT, the Authors suggested that AZA administration in MRD-positive AML patients, might delay disease relapse. Study limitations included the small sample size and the non-randomized design (89). RICAZA trial assessed safety and tolerability of the administration of AZA during the first 12 months after RIC-HCT for adult AML patients. Of the 51 enrolled patients, 37 did receive AZA, starting at a median of 54 days after HCT. A total of 16 patients relapsed at a median time of eight months after HCT. No extensive chronic GvHD was observed. Of note, Authors demonstrated that patients developing a tumor-specific CD8^+^ T cell response experienced a reduced risk of disease relapse (90). CALGB100801 was a phase II single-arm multicenter study exploring the efficacy and tolerability of AZA incorporation within RIC for high risk MDS/AML. A total of 63 patients received a conditioning regimen incorporating targeted Busulfan-based regimen, with ATG for GvHD prophylaxis; Forty-one patients received AZA at 32 mg/m^2^ for 5 days, at a median of 61 days after HCT. NRM and RI at 2 years were 33.4% and 25%, respectively; LFS and OS were 26.9% and 31.2% at 5 years. Grades II-IV and III-IV acute GvHD at 100 days were of 36.5% and 12.7%, respectively. The cumulative incidence of extensive chronic GvHD at 2 years of 14% (91). Colleagues from France reported on a phase II study of the combination of low-dose AZA and DLI. The study enrolled 30 AML and MDS patients with high risk of disease recurrence. AZA began in median 66 days after HCT at the dosage was 32 mg/m2 for five days in a 28-day cycle. DLI were scheduled at escalating doses after cycles 3, 5 and 7. The experimental arm showed an inferior relapse incidence respect to a historical control group, although not statistically significant, with 2-year RI of 27% *vs.* 41% (36). Two different multicenter randomized trials explored the role of HMA as a maintenance treatment for AML/MDS patients after allogeneic HCT. In the first, a total of 187 adult AML or MDS patients were randomized to AZA at dose of 32 mg/m2 for five days in 28-days cycle, up to 12 cycles or observation. The screening failure rate was considerable and time to accrual slow. A total of 24 patients among the 93 patients assigned to maintenance, could complete all the planned twelve cycles; the median time to treatment start was day+63 after HCT. Quite disappointingly, AZA maintenance failed the primary object of the study, with a median LFS of 2 and 1.8 years, for AZA vs. control group, respectively (p = 0.43) (92). The latter was conducted in China and investigated the combination of recombinant human granulocyte-colony stimulating factor with decitabine, to augment the rate of decitabine entry in leukemic cells, as post-HCT maintenance. The Investigators randomized AML patients to receive G-CSF 100 mg/m2 from day 0 to 5 and decitabine 5 mg/m2 on days 1-5 in 6 weeks cycle, up to a maximum of 6 cycles (G-DEC group) or observation only in the control group. A total of 204 high-risk adult AML patients were enrolled. The primary object of the study was met, with 2-year RI of 15% for the experimental arm and 38% in the control group. Of note, in a multivariate model, increased NK cells were associated with a lower relapse incidence (93). The oral formulation of AZA, CC-486, allows for extended DNA demethylation by using longer cycle scheduling (94). De Lima reported results of the prospective multicenter phase I/II dose-finding study of CC-486 maintenance treatment after allogeneic HCT in AML and MDS. A total of 30 adult patients did receive CC-486 at four different dosing schedules in 28 days cycles, starting between 42 to 84 days after HCT. Three patients (10%) experienced grade III acute GvHD and nine chronic GvHD. Of 28 evaluable patients, 6 (21%) relapsed or had progressive disease. At 19 months of follow-up, median overall survival was not reached. The One-year relapse-and LFS rate was 72% with 14-day schedule dosing (95). AMADEUS is a prospective (NCT04173533), two arm, double-blind, phase III clinical trial in allografted patients for AML and MDS, randomized to receive CC-486 or placebo upon engraftment for up to one year as maintenance therapy. Patients will be stratified by type of transplant, myeloablative or RIC, by age (<60/≥ 60 years) and donor type (sibling/unrelated). Estimated completion date is 2025. Primary objective is to assess LFS at one year from randomization of oral AZA compared with placebo. Gaudecitabine (GDC) is a next-generation HMA, with improved pharmacokinetics and greater DNA methylation than decitabine. The phase 2 trial NCT02684162 – is exploring the efficacy of GDC as a mean to control disease relapse after allogeneic HCT. Primary objective of the study is the CR rate of GDC with or without DLI for the treatment of hematologic relapse or MRD in adult AML/MDS patients after transplantation. Interim analysis was recently presented: GDC was given as 30 mg/m2/die for 5 days every 28 until completion of 12 cycles and started between 42 to 100 days after HCT. For 22 treated patients, after a median follow-up of 13.1 months, LFS and OS at 1-year were 66.3% and 88.9%, respectively (96).

### Combinations of HMA with other agents

Combining the polymorphic effects of HMA with novel molecules for disease relapse prevention after allogeneic HCT among AML patients are ongoing. NCT04055844 is a multi-center, single-arm, open-label, phase II trial for the frontline treatment of relapsed AML or MDS following HCT. Enrolled patients will receive up to four cycles of DEC plus JAK2 inhibitor Ruxolitinib and DLI. The Group Francophone del Myélodysplasies is conducting a prospective single-arm phase II trial evaluating the efficacy of a novel product based on the combination of oral decitabine and cedazuridine, a novel cytidine deaminase inhibitor, associated with late DLI after allogenic HCT in very high risk MDS or AML patients (NCT04857645). Panobinostat (PBS) is a histone deacetylase inhibitor, approved for the treatment of multiple myeloma patients with relapsed/refractory disease. Previous experiences demonstrated its efficacy among AML and high risk MDS patients in the context of allogeneic HCT (47). The recent phase I/II trial promoted by the European HOVON consortium, showed the feasibility of PBS after HCT and DLI, although it failed to demonstrate the efficacy of the combination with DEC. Three dose levels were evaluated for dose-limiting toxicities, including PBS monotherapy and PBS combined with two different doses of DEC. A total of 60 patients of the 100 treated went on to receive the DLI. RI was 35%, while OS and LFS were 50% and 49% respectively. Grades II-IV and III-IV Acute GvHD at 6 months were observed in 5% and 23%, respectively; 22% developed moderate-to-severe chronic GvHD at 12 months. Adverse events related to PBS and/or DEC were limited, and hematological grade 3 and 4 events were registered in four patients (48). A European intergroup study is evaluating the efficacy of PBS in poor-risk AML patients after allogeneic HCT (ETAL-4/HOVON145; ECT 2017-000764-15). Anti-Bcl2 Venetoclax (VEN) is a molecule with anti-apoptotic effects. Bcl2 family proteins are overexpressed in AML blasts and their role for tumor survival is essential (97). Colleagues from China recently published their experience with VEN in combination with hypomethylating agent AZA and DLI. A total of 26 adult AML patients relapsing after HCT received the combination of VEN (maximum dose 400 mg/day), AZA at 75 mg/m2 days 1-5 every 28 days for 6-8 cycles and G-CSF stimulated DLI; no patients received cyclosporine to prevent GvHD. All patients developed neutropenia and thrombocytopenia Grades 3 and 4; Six patients developed GvHD. Overall response rate was 61.5%, median LFS and OS were 120 and 284.5 days, respectively (98). A retrospective analysis described the experience with VEN and DLI in 22 patients with AML relapsed after allogeneic HCT. Overall response was observed in eleven patients, with median survival of 6.1 months. Acute GvHD was observed in four patients and chronic GvHD was observed in six patients. Over 50% of patients required hospitalization from infectious events requiring intravenous antibiotics (99). The German Cooperative Transplant Study Group recently presented data from a retrospective analysis conducted on 32 adults with myeloid malignancies relapsing after an allogeneic HCT and treated with HMA and VEN. The patients received VEN (doses between 200 and 800 mg/day) plus AZA (n= 13) or DEC (n= 19) and DLI (n= 11). Median cycle numbers for each patient were two. Overall response rate was 47% (86% in first salvage *vs.* 35% for subsequent lines). After a median follow-up of 8.4 months, seven patients were alive. Infections requiring hospitalization were a frequent event (72%) and myelotoxicity was observed for almost all patients. One patient developed chronic GvHD (100). A phase I multicenter single-arm trial has just opened enrollment for adult AML patients with TP53 mutation, evaluating safety and efficacy of DEC plus VEN, followed by DEC maintenance at dose of 5mg/m2 for 5 consecutive days every 6-8 weeks, for a total of 4 or 6 courses and DLI in case of MRD positivity. (NCT05528354). Given its immunomodulatory properties, Lenalidomide (LEN) could be a promising strategy in the maintenance setting following allogeneic HCT; However, it has been associated with high rates of severe and life-threatening GvHD, resulting in a strong contraindication in such a setting (101). Given the favorable profile played by AZA respect to Treg population reconstitution soon after HCT, Investigators have explored the combination of the two molecules (102). A complex dose-finding study of LEN and AZA (VIOLA trial), showed that LEN could be administered safely at proper dosage after allografting, without notable clinical efficacy. Twenty-nine AML/MDS adult patients relapsing after HCT were treated with AZA at 75 mg/m2 for seven days followed by LEN in escalating doses. The combination was well tolerated, with a maximum dose of LEN of 25 mg/day. Three patients developed GvHD at 15 mg daily dose, two at 25 mg/day; all patients responded to steroid treatment and there were no GvHD-related deaths. Seven out of 15 patients achieve a major clinical response (103). Investigators from Germany explored the tolerability and efficacy of the combination of AZA, LEN and DLI as first salvage therapy for AML, MDS and chronic myelomonocytic leukemia relapsing after allogeneic HCT in a prospective, multicenter, single-arm phase-II trial (AZALENA trial). A total of 50 patients received AZA 75 mg/m2 days 1-7, every 28 days, LEN 2.5 mg/daily for 21 consecutive days of a 28-day cycle for the first 10 patients, then switched to 5 mg/daily for the remaining, and DLI, with increasing dose, for a maximum of 3 infusions. Overall response rate was 56%, with a median time to best response of 112 days. A total of twenty patients with CR received DLI. After a median follow-up of 20 months, median OS was 21 months and 1-year OS rate 65%, with 80% of patients in CR at last follow-up. The rate of GvHD was not negligible, with 23 patients developing acute GvHD including five with grade III/IV, and 26 chronic GvHD (moderate n=11; severe n=5) (104).

## FLT3-inhibitors

FMS-like tyrosine kinase 3 (FLT3) is the most frequent single-gene mutation in acute myeloid leukemia: about 25% of adult patients with AML have FLT3 internal tandem duplication (FLT3-ITD) and 10% have FLT3 tyrosine kinase domain (FLT3-TKD) point mutations or deletions (105). FLT3 inhibitors include first generation agents, non-specific multi-kinase inhibitors, targeting not only FLT3, such as sorafenib and midostaurin, and second generation molecules such as quizartinib, gilteritinib and crenolanib, that are more potent and selective agents.

### Sorafenib

Sorafenib was the first agent used in the transplant setting, based on early observations of prolonged survival rates among FLT3-ITD mutated AML relapsing after HCT (106). In 2020 two randomized placebo-controlled trials were published, testing the efficacy of Sorafenib in inhibiting FLT3-ITD–positive AML recurrence after HCT: SORMAIN in Europe and the study by Xuan et al. in China. SORMAIN was a phase II, multicenter, randomized, double-blind, placebo-controlled trial, comparing sorafenib versus placebo as prophylactic treatment after HCT. It enrolled a total of 83 patients in CR after HCT. Patients were randomly assigned 1:1 to receive either Sorafenib (n=43) or Placebo (n=40) for 24 months or until occurrence of relapse or intolerable toxicity, starting between day +60 to day +100 after HCT. The study met its primary endpoint: the estimated probability of 24-month LFS was 85% in the experimental arm and 53.3% in the placebo group, corresponding to an HR for relapse or death of 0.256. At a median follow-up of 41.8 months, the median LFS was not reached for sorafenib group. This study was the first to provide placebo-controlled evidence that post-HCT maintenance therapy can reduce the risk of relapse and death (107). However, it enrolled fewer patients than originally planned by the study design, mostly because many patients received maintenance therapy with sorafenib, off label outside of a clinical trial, based on results from previous uncontrolled retrospective studies (108, 109) showing efficacy and feasibility of sorafenib after allogeneic HCT, and expert recommendations (110). The second trial enrolled a total of 202 patients, with a relatively young median age of 35 years, in CR after HCT, randomly assigned (1:1) to receive either Sorafenib (n=100) or no maintenance (control group, n=102), starting from day between +30 to +60 after HCT, until day +180. The initial dose of Sorafenib was 400 mg twice daily. At a median follow up of 21.3 months,11 patients in the sorafenib group and 32 in the control group relapsed. The median time to relapse was 11.6 months after transplant among patients assigned to sorafenib and 5.7 months in those allocated to control. The most common serious adverse events in both groups were: infections (25% similar in both groups), acute GvHD (23% in Sorafenib and 21% in control group), chronic GvHD (18% in Sorafenib vs. 17% in controls) (111). The rate of GvHD was not increased by the administration of Sorafenib, suggesting that GvL effect can occur and synergize with the drug independently from GvHD. In fact, experimental observations highlighted the biological base of this intuition. Researchers showed that Sorafenib increased IL-15 production by FLT3-ITD leukemia cells, which in turn caused an increase in CD8+CD107a+IFN y T cells which eradicated leukemia in secondary recipients. FLT3-ITD^+^ AML cells obtained from sorafenib responders showed increased IL-15 levels, a strong activator of allogenic T cell responses (112). However, many open issues remain to be addressed: the optimal time of starting and duration of therapy, the optimal dosing, the use of sorafenib in patients previously exposed to midostaurin before HCT, the use of sorafenib *vs.* 2^nd^ generation FLT3-inhibitors. The theoretical concern of selecting residual FLT-wild type subclones or the induction of drug-resistance, through the acquisition of novel mutations in TKD domain, represents a matter of debate. In SORMAIN trial, 4 out of 10 disease relapses occurred after the end of sorafenib retreatment and might be preventable by longer maintenance duration. On the other hand, in Chinese trial, of the 43 patients who relapsed, five of 11 assigned sorafenib and 17 of 32 allocated control had FLT3-ITD mutations, one patient assigned sorafenib acquired an FLT3 tyrosine kinase domain (FLT3-TKD) mutation at relapse; The other twenty patients were FLT3 wild type at relapse. In the recent EBMT consensus (113) the recommended dose of sorafenib maintenance post-transplantation is 200 mg twice a day for patients without minimal residual disease and 400 mg twice a day for patients with minimal residual disease. The duration of maintenance therapy is not firmly established, but a minimum of 2 years is recommended, depending on tolerance. Investigators from Dusseldorf presented some years ago their experience with AZA and Sorafenib as salvage therapy for FLT3-ITD mutated AML after HCT. Eight relapsed patients were treated with a median of five cycles of AZA, Sorafenib and, in six patients, DLI (median of two infusions). Four patients reached a CR, with a median duration of CR of 182 days; Median OS was 322 days (114). The NCT04674345 is a phase 2 randomized multicenter prospective study from China aiming at evaluating the role of Sorafenib maintenance after HCT among FLT3-negative AML patients.

### Midostaurin

A first-generation type I inhibitor, active on both FLT3-ITD and TKD mutants, Midostaurin proved to be efficacious in improving the survival rate of FLT3-mutated AML patients, if added to standard chemotherapy. The phase II trial AMLSG 16-10 evaluated the addition of midostaurin to intensive chemotherapy followed by allogeneic HCT and single agent maintenance therapy of 12 months in patients aged 18-70 years with FLT-ITD AML. Maintenance therapy was started in 97 patients. LFS was compared to historical controls and seemed to be significantly improved by midostaurin (115). Maziarz et al. specifically evaluated the role of midostaurin maintenance after allogenic HCT in patients with FLT3-ITD positive AML, transplanted in first CR in a phase 2, randomized, open-label trial (RADIUS) that compared standard of care (SOC) with or without midostaurin. Patients were enrolled after engraftment and randomized (1:1) to receive SOC +/- midostaurin. There was a trend to a better LFS in midostaurin group, but not statistically significant: the estimated LFS at 18 months was 89% with midostaurin and 76% with SOC alone Only 60 patients, 30 per arm, were randomized and half of them completed the 12 cycles. Moreover, the LFS in SOC (76%) was better than expected (about 60%), maybe because of the stringent inclusion criteria, this may have underestimated the difference between the two arms (116). The drug was generally well tolerated in both studies^;^ Of note, the addition of midostaurin to SOC did not increase the rate or severity of acute or chronic GvHD. Overall, the evidence to recommend the use of midostaurin after allogenic HCT is still limited and the drug is not approved for this indication.

### Gilteritinib

A highly selective, 2^nd^ generation oral FLT3 type I inhibitor with activity against both FLT3 mutation subtypes (ITD and TKD) and weak activity against c-Kit, Gilteritinib (117) has been approved by EMA for treatment of relapsed/refractory AML with FLT3-ITD or TKD mutations, on the basis of the results of a phase III trial (118). ADMIRAL trial enrolled 371 patients with relapsed/refractory FLT3-mutated (ITD or TKD) AML, randomly assigned (2:1) to receive either gilteritinib (120 mg per day) or salvage chemotherapy. Differently from RATIFY trial, responders’ patients could go on and receive an allogeneic HCT and resume gilteritinib therapy 30 to 90 days after the transplantation if they had engraftment without relapse and no uncontrolled complications. Based on this study design, gilteritinib is the only FLT3-ITD with a label indication after transplant in Europe. It is important to note that, even if previous treatment with sorafenib or midostaurin was allowed in the study, most patients enrolled were not exposed to a FLT3-inhibitor and only 5.7% of them had received midostaurin. Previous exposure to midostaurin could plausibly generate resistance to FLT3-targeted therapy and subsequently alter gilteritinib activity. To address this question, an ongoing trial (NCT02997202, MORPHO) has been specifically designed, comparing Gilteritinib to placebo as maintenance therapy after HCT for patients in first CR, from engraftment to 24 months after HCT.

## Novel molecules

The CTLA-4 (CD152) receptor inhibits T-cell maturation and differentiation by competing with the costimulatory receptor CD28 for antigen presenting cells CD80 and CD86, as well as PD-1 expression by T-cells is a known mechanism of anergy (119). The inhibition of CTLA-4/B7 or PD-1/PDL-1 axis may restore immune surveillance (120). Ipilimumab is an anti-CTLA-4 antibody that binds CTLA-4 on T cells (121). In AML, there is an increased expression of CD80 and CD86 on leukemic blasts, thus the inhibition of CTLA-4 may restore immune reaction against tumor, mostly in the context of GvL (122). This hypothesis has been tested in a phase I trial, that enrolled patients with relapsed hematologic cancer after allogeneic HCT, in which 14 patients with AML/MDS were involved (123). CR occurred in four patients; Four patients had a durable response for more than one year. Responses were associated with *in situ* infiltration of cytotoxic CD8+ T cells, decreased activation of regulatory T cells, and expansion of subpopulations of effector T cells in the blood. Dose-limiting toxic effects included two cases of chronic GvHD of the liver and one case of grade II acute GvHD of the gut, all of which resolved with glucocorticoids. The multicenter phase I trial (CTEP 10026) of DEC plus ipilimumab in patients with relapse/refractory AML/MDS is testing safety and efficacy of the combination. Sixteen patients have been enrolled in the allogeneic arm trial. One patient developed late-onset acute grade 3 GvHD, while chronic GvHD developed in four patients. One patient died from GvHD complications. Four patients out of 16 obtained CR. Median OS was 12.8 months (124).

Mutations in isocitrate dehydrogenase (IDH)*1/2* genes promote leukemogenesis and impair normal myeloid differentiation and are generally seen in approximately 20% of AML patients (125). A retrospective report from City of Hope demonstrated that, in a cohort of 99 allografted patients, IDH mutation was the only predictor of disease relapse after HCT in multivariate analysis (126). Based on this data, several clinical trials are testing safety and efficacy of IDH inhibitors ivosidenib/edasidenib in the maintenance phase after HCT: NCT03515512 is a Phase 1 trial assessing the safety and efficacy of Evosidenib for relapse prevention of IDH2 mutated AML or CMML adult patients after allogeneic HCT; NCT04522895 is a German-based prospective, single arm, multicenter phase 2 trial aiming to evaluate efficacy of Enasidenib as maintenance therapy after HCT for myeloid neoplasms; NCT03728335 is a phase 1 trial investigating the role of Enasidenib as maintenance therapy in AML with IDH2 mutation after HCT.

MDM2 inhibition can contrast AML post-HCT immunological escape through the restoration of TRAIL-R1/2 and class 2 MHC production (127). MDM2 inhibition enhances cytotoxic effects against leukemic cells, leading to an increase in p53-dependent tumor necrosis factor-related apoptosis-inducing ligand receptor-1/2 (TRAIL 1/2) and MHC-class II on blast cells and higher levels of T cells with cytotoxic features (128). NCT05447663 – is a Phase Ib/II, single arm, open label, multi-center study of siremadlin, as monotherapy and in combination with DLI, in adult participants with high-risk AML in CR after allogeneic HCT.

T cell immunoglobulin and mucin domain-containing protein 3 (TIM3) is a glycoprotein expressed on the surface of Treg, NK cells, macrophages, dendritic and myeloid cells. It is typically upregulated on AML cells (32). TIM3 inhibitors are capable of directly inhibiting leukemic cells and restoring T-cells function from exhaustion state (129). Results from a multi-arm, open-label phase 1 study conducted on 28 MDS/AML patients, receiving escalated doses of Sabatolimab plus DEC or AZA showed encouraging results, with 2-year OS and LFS of 69% and 64%, respectively. Fifty-seven percent of patients experienced acute GvHD, with 14% of grade 3/4 acute GvHD and 29% of chronic GvHD requiring immunosuppressive therapy (130). NCT04623216 is a phase 1-2 trial testing the role of preemptive TIM3 inhibitor Sabatolimab, alone or in combination with AZA, for MRD-positive AML patients after HCT (**Table 3**).

**Table 3:** Active Clinical trials in post-HCT relapse of AML/MDS.

Study	Phase	No.patients	Intervention	Setting	Type
NCT03300492	Phase 1/2	10	Expanded NK cells in haploidentical HCT	Prophylactic	Prospective
NCT03597321	Phase 2	124	Early DLI after HCT	Prophylactic	Randomized
NCT02452697	Phase 2	100	NK-DLI +/- TLR9 agonist	Prophylactic	Randomized
NCT03939585	Phase 1	10	TCR-αβ T cells and B cells	Prophylactic	Single group assignment
NCT04173533	Phase 3	324	Oral AZA vs. Placebo	Prophylactic	Randomized
NCT02684162	Phase 2	55	Guadecitabine + DLI	Post-HCT relapse	Single group assignment
NCT04055844	Phase 2	14	Ruxolitinib+DEC+DLI	Post-HCT relapse	Multi-centre, single arm
NCT04857645	Phase 2	40	ASTX727+DLI	Prophylactic	Prospective
NCT05528354	Phase 1	58	VEN+DEC conditioning and DEC maintenance	Prophylactic	Multi-centre, single arm
NCT04674345	Phase 2/3	346	Sorafenib	Prophylactic	Multi-centre, prospective
NCT02997202	Phase 3	356	Gilteritinib	Prophylactic	Randomized, double-blind
NCT03515512	Phase 1	23	Evosidenib	Prophylactic	Single group assignment
NCT04522895	Phase 2	50	Enasidenib	Prophylactic	Multi-centre, prospective
NCT03728335	Phase 1	15	Enasidenib	Prophylactic	Single group assignment
NCT05447663	Phase 1/2	32	Siremadlin+/- DLI	Prophylactic	Single group assignment
NCT04623216	Phase 1/2	59	Sabatolimab	Treatment MRD	Randomized

HCT, hematopoietic cell transplantation; RIC, reduced-intensity conditioning; MAC, myeloablative conditioning; VE; venetoclax; DEC, decitabine; AZA, azacytidine; MRD, minimal residual disease.

## Conclusions and perspectives

The Holy grail of allogeneic transplantation lies in the possibility of separate GvHD from GvL effects. The success of HCT is largely limited by disease relapse. The prevention of relapse starts directly in the delicate phase of remission induction therapies, as the absence of minimal-residual disease prior to HCT provides the best chance of cure. Although relapse reflects the failure of conditioning regimen to ablate recipient tumor cells, it also critically depends upon the absence, or the ineffectiveness of a specific donor T-cell mediated immunologic reaction. GvL responses and their manipulation for therapeutic scopes are currently being explored as a rationale, biological countermeasure to face the relapse problem. DLI represent one of the most used strategies aiming to intensify GvL effect. With novel biological insights into AML relapse mechanisms, combinatorial treatments of adoptive immunotherapies with target-therapies and novel molecules are currently being tested for safety and efficacy in the difficult post-transplant relapse scenario. Efforts to separate potentially lethal effects of GvHD from desired GvL effects will continue to dominate both biological and clinical studies in the future. As Heraclitus sharply said: “What is opposition is reconciled and from different things, the most beautiful harmony is born, and everything is generated through contrast”.

## Author contributions

EM and MU wrote the manuscript. FBo critically revised the manuscript. EM and FBo edited the final version of the manuscript All authors contributed to the article and approved the submitted version.

## Funding

The work reported in this publication was funded by the Italian Ministry of Health, RC-2022-2773290.

## Acknowledgments

The authors thank AIL Bologna ODV, the Italian Association for research on leukemia, lymphoma and myeloma, for the support of the Laboratory of Immunobiology of Transplant and Cellular Therapies, IRCCS AOU Bologna; Bologna, Italy, led by Francesca Bonifazi, MD, PhD.

## Conflict of interest

The authors declare that the study was conducted in the absence of any commercial or financial relationship deemed to be a potential conflict of interest.

## Publisher’s note

All claims expressed in this article are solely those of the authors and do not necessarily represent those of their affiliated organizations, or those of the publisher, the editors and the reviewers. Any product that may be evaluated in this article, or claim that may be made by its manufacturer, is not guaranteed or endorsed by the publisher.

## References

[1] Horowitz M Schreiber H Elder A Heidenreich O Vormoor J Toffalori C . Epidemiology and biology of relapse after stem cell transplantation. Bone Marrow Transplant (2018) 53:1379–89. doi: 10.1038/s41409-018-0171-z PMC6282701 29670211

[2] De Jong G Janssen JJ Biemond BJ Zeerleder SS Ossenkoppele GJ Visser O . Survival of early post hematopoietic stem cell transplantation relapse of myeloid malignancies. Eur J Hematol (2019) 103:491–9. doi: 10.1111/ejh.13315 PMC6851577 31411761

[3] Bejanyan N Weisdorf DJ Logan BR Wang HL Devine SM de Lima M . Survival of patients with acute myeloid leukemia relapsing after allogeneic hematopoietic cell transplantation: A center for international blood and marrow transplant research study. Biol Blood Marrow Transplant (2015) 21(3):454–9. doi: 10.1016/j.bbmt.2014.11.007 PMC4329076 25460355

[4] Mielcarek M Storer BE Flowers ME Storb R Sandmaier BM Martin PJ . Outcomes among patients with recurrent high-risk hematologic malignancies after allogeneic hematopoietic cell transplantation. Bio Blood Marrow Transplant (2007) 13:1160–8. doi: 10.1016/j.bbmt.2007.06.007 17889352

[5] Yanada M Konuma T Yamasaki S Kondo T Fukuda T Shingai N . Relapse of acute myeloid leukemia after allogeneic hematopoietic cell transplantation: clinical features and clinical outcomes. Bone Marrow Transplant (2021) 56:1126–33. doi: 10.1038/s41409-020-01163-z 33268829

[6] Brambilla Zuanelli C Lobaugh SM Ruiz JD Dahi PB Goldberg AD Young JW . Relapse after allogeneic stem cell transplantation of acute myelogenous leukemia and myelodysplastic syndrome and the importance of second cellular therapy. Transplant Cell Ther (2021) 27(9):771.e1–771.e10. doi: 10.1016/j.jtct.2021.05.011 PMC9236560 34033977

[7] Ossenkoppele GJ Janssen JJ van de Loosdrecht AA . Risk factors for relapse after allogeneic transplantation in acute myeloid leukemia. Haematologica (2016) 101:20–5. doi: 10.3324/haematol.2015.139105 PMC4697888 26721801

[8] Thanarajasingam G Kim HT Cutler C Ho VT Koreth J Alyea AP . Outcome and prognostic factors for patients who relapse after allogeneic hematopoietic stem cell transplantation. Biol Blood Marrow Transplant (2013) 19:1713–8. doi: 10.1016/j.bbmt.2013.09.011 PMC3848699 24076323

[9] Schuler E Boughoufala S Rautenberg C Nachtkamp K Dienst A Fenk R . Relapse patterns and treatment strategies in patients receiving allogeneic hematopoietic stem cell transplantation for myeloid malignancies. Ann Hematol (2019) 98:1225–35. doi: 10.1007/s00277-019-03670-6 30923997

[10] Zeiser R Vago L . Mechanisms of immune escape after allogeneic hematopoietic cell transplantation. Blood (2019) 133(12):1290–7. doi: 10.1182/blood-2018-10-846824 30578254

[11] Vago L Perna SK Zanussi M Mazzi B Barlassina C Lupo Stanghellini MT . Loss of mismatched HLA in leukemia after stem-cell transplantation. N Eng J Med (2009) 361:478–88. doi: 10.1056/NEJMoa0811036 19641204

[12] Crucitti L Crocchiolo R Toffalori C Mazzi B Greco R Signori A . Incidence, risk factors and clinical outcome of leukemia relapses with loss of the mismatched HLA after partially incompatible hematopoietic stem cell transplantation. Leukemia (2015) 29:1143. doi: 10.1038/leu.2014.314 25371177

[13] Shyr DC Zhang BM Saini G Madani ND Schultz LM Patel S . HLA-haplotype loss after TCR αβ/CD19-depleted haploidentical HSCT. Bone Marrow Transplant (2021) 56:733–7. doi: 10.1038/s41409-020-01081-0 33070150

[14] Vago L Toffalori C Ahci M Lange V Lang K Todaro S . Incidence of HLA loss in a global multicentric cohort of post-transplantation relapses: Results from the hlaloss collaborative study. Blood (2018) 132:818. doi: 10.1182/blood-2018-99-112142

[15] Adachi Y Sakai T Terakura S Shiina T Suzuki S Hamana H . Downregualtion of HLA class II is associated with relapse after allogeneic stem cell transplantation and alters recognition by antigen-specific T cells. Int J Hematol (2022) 115(3):371–81. doi: 10.1007/s12185-021-03273-w 35037229

[16] Cristopher JM Petti AA Rettig MP Miller CA Chendamarai E Duncavage EJ . Immune escape of relapsed AML cells after allogeneic transplantation. N Eng J Med (2018) 379:2330–41. doi: 10.1056/NEJMoa1808777 PMC6322675 30380364

[17] Jan M Leventhal MJ Morgan EA Wengrod JC Nag A Drinan SD . Recurrent genetic HLA loss in AML relapsed after matched unrelated allogeneic hematopoietic cell transplantation. Blood Adv (2019) 3(14):2199–204. doi: 10.1182/bloodadvances.2019000445 PMC6650729 31324640

[18] Toffalori C Zito L Gambacorta V Riba M Oliveira G Bucci G . Immune signature drives leukemia escape and relapse after hematopoietic cell transplantation. Nat Med (2019) 25(4):603–11. doi: 10.1038/s41591-019-0400-z 30911134

[19] Gambacorta V Beretta S Ciccimarra M Zito L Giannetti K Andrisani A . Integrated multiomic profiling identifies the epigenetic regulator PRC2 as a therapeutic target to counteract leukemia immune escape and relapse. Cancer Discovery (2022) 12(6):1449–61. doi: 10.1158/2159-8290.CD-21-0980 PMC9394393 35255120

[20] Hutten TJA Norde WJ Woestenenk R Wang RC Maas F Kester M . Increased coexpression of PD-1, TIGIT, and KLRG-1 on tumor-reactive CD8+ T cells during relapse after allogeneic stem cell transplantation. Biol Blood Marrow Transplant (2018) 24(4):666–77. doi: 10.1016/j.bbmt.2017.11.027 29197680

[21] Naka K Hoshii T Muraguchi T Tadokoro Y Ooshio T Kondo Y . TGF-beta-FOXO signalling maintains leukaemia-initiating cells in chronic myeloid leukaemia. Nature (2010) 463(7281):676–80. doi: 10.1038/nature08734 20130650

[22] Lee YJ Han Y Lu HT Nguyen V Qin H Howe PH . TGF-beta suppresses IFN-gamma induction of class II MHC gene expression by inhibiting class II transactivator messenger RNA expression. J Immunol (1997) 158(5):2065–75. doi: 10.4049/jimmunol.158.5.2065 9036950

[23] Romee R Cooley S Berrien-Elliott MM Westervelt P Verneris MR Wagner JE . First-in-human phase 1 clinical study of the IL-15 superagonist complex ALT-803 to treat relapse after transplantation. Blood (2018) 131(23):2515–27. doi: 10.1182/blood-2017-12-823757 PMC5992862 29463563

[24] Jacoby MA Duncavage EJ Chang GS Miller CA Shao J Elliott K . Subclones dominate at MDS progression following allogeneic hematopoietic cell transplant. JCI Insight (2018) 3(5):98962. doi: 10.1172/jci.insight.98962 29515031 PMC5922277

[25] Quek L Ferguson P Metzner M Ahmed I Kennedy A Garnett C . Mutational analysis of disease relapse in patients allografted for acute myeloid leukemia. Blood Adv (2016) 1(3):193–204. doi: 10.1182/bloodadvances.2016000760 29296935 PMC5737177

[26] Kolb HJ Mittermuller J Clemm C Holler E Ledderose G Brehm G . Donor leukocyte transfusions for treatment of recurrent chronic myelogenous leukemia in marrow transplant patients. Blood (1990) 76:2462–5. doi: 10.1182/blood.V76.12.2462.2462 2265242

[27] Kolb HJ Schattenberg A Goldman JM Hertenstein B Jacobsen N Arcese W . Graft-versus-leukemia effect of donor lymphocyte transfusions in marrow grafted patients. Blood. (1995) 86:2041–50. doi: 10.1182/blood.V86.5.2041.bloodjournal8652041 7655033

[28] Kolb HJ Schmid C Barrett AJ Schendel DJ . Graft-versus-leukemia reactions in allogeneic chimeras. Blood. (2004) 103:767–76. doi: 10.1182/blood-2003-02-0342 12958064

[29] Collins RH Shpilberg O Drobyski WR Porter DL Giralt S Champlin R . Donor leukocyte infusions in 140 patients with relapsed malignancy after allogeneic bone marrow transplantation. J Clin Oncol (1997) 15:433–44. doi: 10.1200/JCO.1997.15.2.433 9053463

[30] Liu L Chang YJ Xu LP Zhang XH Wang Y Liu KY . Reversal of T cell exhaustion by the first donor lymphocyte infusion is associated with the persistently effective antileukemic responses in patients with relapsed AML after allo-HSCT. Biol Blood Marrow Transplant (2018) 24:1350–9. doi: 10.1016/j.bbmt.2018.03.030 29649617

[31] Bachireddy P Azizi E Burdziak C Nguyen VN Ennis CS Maurer K . Mapping the evolution of T cell states during response and resistance to adoptive cellular therapy. Cell Rep (2021) 37(6):109992. doi: 10.1016/j.celrep.2021.109992 34758319 PMC9035342

[32] Kong Y Zhang J Claxton DF Ehmann WC Rybka WB Zhu L . PD-1(hi)TIM-3(+) T cells associate with and predict leukemia relapse in AML patients post allogeneic stem cell transplantation. Blood Cancer J (2015) 5(7):e330. doi: 10.1038/bcj.2015.58 26230954 PMC4526784

[33] Noviello M Manfredi F Ruggiero E Perini T Oliveira G Cortesi F . Bone marrow central memory and memory stem T-cell exhaustion in AML patients relapsing after HSCT. Nat Commun (2019) 10(1):1065. doi: 10.1038/s41467-019-08871-1 30911002 PMC6434052

[34] Van Bergen CAM Van Luxemburg-Heijs SAP De Wreede LC Eefting M Von Dem Borne PA Van Balen P . Selective graft-versus-leukemia depends on magnitude and diversity of the alloreactive T cell response. J Clin Invest (2017) 127:517–29. doi: 10.1172/JCI86175 PMC5272193 28067665

[35] Schultze-Florey CR Kuhlmann L Raha S Barros-Martins J Odak I Tan L . Clonal expansion of CD8+ T cells reflects graft-versus-leukemia activity and precedes durable remission following DLI. Blood Adv (2021) 5:4485–99. doi: 10.1182/bloodadvances.2020004073 PMC8579265 34535011

[36] Guillame T Malard F Magro L Labopin M Tabrizi R Borel C . Prospective phase II study of prophylactic low-dose azacitidine and donor lymphocyte infusions following allogeneic hematopoietic stem cell transplantation for high-risk acute myeloid leukemia and myelodysplastic syndrome. Bone Marrow Transplant (2019) 54(11):1815–26. doi: 10.1038/s41409-019-0536-y 31089280

[37] Dominietto A Pozzi S Miglino M Albarracin F Piaggio G Bertolotti F . Donor lymphocyte infusions for the treatment of minimal residual disease in acute leukemia. Blood (2007) 109:5063–4. doi: 10.1182/blood-2007-02-072470 17522340

[38] Mo XD Zhang XH Xu LP Wang Y Yan CH Chen H . Salvage chemotherapy followed by granulocyte colony-stimulating factor-primed donor leukocyte infusion with graft-vs-Host disease control for minimal residual disease in acute Leukemia/Myelodisplastic syndrome after allogeneic hematopoietic stem cell tran. Eur J Haematol (2016) 96:297–308. doi: 10.1111/ejh.12591 26010204

[39] Schmid C Schleuning M Ledderose G Tischer J Kolb HJ . Sequential regimen of chemotherapy, reduced-intensity conditioning for allogeneic stem-cell transplantation, and prophylactic donor lymphocyte transfusion in high-risk acute myeloid leukemia and myelodysplastic syndrome. J Clin Oncol (2005) 23:5675–87. doi: 10.1200/JCO.2005.07.061 16110027

[40] Jedlickova Z Schmid C Koenecke C Hertenstein B Baurmann H Schwerdtfeger R . Long-term results of adjuvant donor lymphocyte transfusion in AML after allogeneic stem cell transplantation. Bone Marrow Transplant (2016) 51:663–7. doi: 10.1038/bmt.2015.234 26437060

[41] Mohty M Malard F Blaise D Milpied N Sociè G Huynh A . Sequential regimen of clofarabine, cytosine arabinoside and reduced-intensity conditioned transplantation for primary refractory acute myeloid leukemia. Haematologica (2017) 102:184–91. doi: 10.3324/haematol.2016.150326 PMC5210249 27561720

[42] Gilman AL Leung W Cowan MJ Cannon M Epstein S Barnhart C . Donor lymphocyte infusion and methotrexate for immune recovery after T-cell depleted haploidentical transplantation. Am J Haematol (2018) 93(2):169–78. doi: 10.1002/ajh.24949 29047161

[43] Cauchois R Castagna L Pagliardini T Harbi S Calmels B Bramanti S . Prophylactic donor lymphocyte infusions after haploidentical haematopoietic stem cell transplantation for high risk haematological malignancies: A retrospective bicentric analysis of serial infusions of increasing doses of CD3+ cells. Br J Haematol (2019) 185:570–3. doi: 10.1111/bjh.15544 30125934

[44] Yang L Tan Y Shi J Zhao Y Yu J Hu Y . Prophylactic modified donor lymphocyte infusion after low-dose ATG-f-based haploidentical HSCT with myeloablative conditioning in high-risk acute leukemia: a matched pair analysis. Bone Marrow Transplant (2021) 56(3):664–72. doi: 10.1038/s41409-020-01088-7 33077902

[45] Sheth V Potter V de Lavallade H Gandhi S Kulasekararaj Krishnamurthy P . Mixed T cell lineage chimerism in acute leukemia/MDS using pre-emptive donor lymphocyte infusion strategy-is it prognostic?- a single-center retrospective study. Blood Cancer J (2021) 11(7):128. doi: 10.1038/s41408-021-00519-y 34253713 PMC8275738

[46] Zhang R Wang L Chen P Gao X Wang S Li F . Haematologic malignancies with unfavourable gene mutations benefit from donor lymphocyte infusion with/without decitabine for prophylaxis of relapse after allogeneic HSCT: A pilot study. Cancer Med (2021) 10(10):3165–76. doi: 10.1002/cam4.3763 PMC8124122 33932107

[47] Bug G Burchert A Wagner EM Kroger N Berg T Guller S . Phase I/II study of the deacetylase inhibitor panobinostat after allogeneic stem cell transplantation in patients with high-risk MDS or AML (PANOBEST trial). Leukemia. (2017) 31(11):2523–5. doi: 10.1038/leu.2017.242 PMC5668491 28751769

[48] Kalin B van Norden Y van Gelder M Breems D Maertens J Jongen-Lavrencic M . Panobinostat and decitabine prior to donor lymphocyte infusion in allogeneic stem cell transplantation. Blood Adv (2020) 4(18):4430–7. doi: 10.1182/bloodadvances.2020002074 PMC7509859 32936907

[49] Rettig AR Ihorst G Bertz H Lubbert M Marks R Waterhouse M . Donor lymphocyte infusions after first allogeneic hematopoietic stem-cell transplantation in adults with acute myeloid leukemia: A single-center landmark analysis. Ann Hematol (2021) 100:2339–50. doi: 10.1007/s00277-021-04494-z PMC8357755 33796897

[50] Kryshnamurthy P Potter VT Barber LD Kulasekararaj AG Lim ZY Pearce RM . Outcome of donor lymphocyte infusion after T cell-depleted allogeneic hematopoietic stem cell transplantation for acute myelogenous leukemia and myelodysplastic syndromes. Biol Blood Marrow Transplant (2013) 19(4):562–8. doi: 10.1016/j.bbmt.2012.12.013 23266740

[51] Yang L Luo Y Huang H Tan Y Shi J Zhao Y . Comparison of prophylactic and preemptive donor lymphocyte infusion in patients with very high risk acute leukemia after allogeneic hematopoietic stem cell transplantation. Blood (2021) 138(s1):3860. doi: 10.1182/blood-2021-152578

[52] Schmid C Labopin M Nagler A Bornhauser M Finke J Fassas A . Donor lymphocyte infusion in the treatment of first hematological relapse after allogeneic stem-cell transplantation in adults with acute myeloid leukemia: a retrospective risk factors analysis and comparison with other strategies by the EBMT acute leukemia working party. J Clin Oncol (2007) 25:4938–45. doi: 10.1200/JCO.2007.11.6053 17909197

[53] Dholaria B Savani BN Labopin M Luznik L Ruggeri A Mielke S . Clinical applications of donor lymphocyte infusion from a HLA-haploidentical donor: consensus recommendation from the acute leukemia working party of the EBMT. Haematologica (2020) 105(1):47–58. doi: 10.3324/haematol.2019.219790 31537691 PMC6939532

[54] Schmid C Labopin M Schaap N Veelken H Brecht A Stadler M . Long-term results and GvHD after prophylactic and preemptive donor lymphocyte infusion after allogeneic stem cell transplantation for acute leukemia. Bone Marrow Transplant (2022) 57:215–23. doi: 10.1038/s41409-021-01515-3 PMC8821014 34750562

[55] Solomon SR Sizemore CA Zhang X Brown S Holland HK Morris LE . Premptive DLI without withdrawal of immunosuppression to promote complete donor T-cell chimerism results in favourable outcomes for high-risk older recipients of alemtuzumab-containing reduced-intensity unrelated donor allogeneic transplant: A prospective. Bone Marrow Transplant (2014) 49:616–21. doi: 10.1038/bmt.2014.2 24801098

[56] Gilleece MH LAbopin M Yakoub-Agha I Volin L Socié G Ljungman P . Measurable residual disease, conditioning regimen intensity, and age predict outcomes of allogeneic hematopoietic cell transplantation for acute myeloid leukemia in first remission: A registry analysis of 2292 patients by the acute leukemia working party. Am J Hematol (2018) 93:1142–52. doi: 10.1002/ajh.25211 29981272

[57] Tsirigotis P Byrne M Schmid C Baron F Ciceri F Esteve J . Relapse of AML after hematopoietic stem cell transplantation: methods of monitoring and preventive strategies. a review from the ALWP of the EBMT. Bone Marrow Transplant (2016) 51:1431–8. doi: 10.1038/bmt.2016.167 27295272

[58] Tan Y Du K Luo Y Shi J Cao L Zheng Y . Superiority of preemptive donor lymphocyte infusion based on minimal residual disease in acute leukemia patients after allogeneic hematopoietic stem cell transplantation. Transfusion (2014) 54(6):1493–500. doi: 10.1111/trf.12524 24372162

[59] Hussain SA Khan AN Thammineni VS Nauman Riaz M Shahzad M Pulipati P . Outcomes with preemptive donor lymphocyte infusions after allogeneic hematopoietic stem cell transplantation for acute myeloid leukemia and myelodysplastic syndromes: A systematic review and meta-analysis. J Clin Oncol (2021) 39(15):e19014. doi: 10.1200/JCO.2021.39.15_suppl.e19014

[60] Jaiswal SR Bhakuni P Zaman S Bansal S Bharadwaj P Bhargava S . T Cell costimulation blockade promotes transplantation tolerance in combination with sirolimus and post-transplantation cyclophosphamide for haploidentical transplantation in children with severe aplastic anemia. Transpl Immunol (2017) 43-44:54–9. doi: 10.1016/j.trim.2017.07.004 28802588

[61] Santoro N Mooyaart JE Devillier R Koc Y Vydra J Castagna L . Donor lymphocyte infusions after haploidentical stem cell transplantation with PTCY: A study on behalf of the EBMT cellular therapy & immunobiology working party. Bone Marrow Transplant (2022). doi: 10.1038/s41409-022-01839-8 36216975

[62] Greiner J Gotz M Bunjes D Hofmann S Wais V . Immnulogical and clinical impact of manipulated and unmanipulated DLI after allogeneic stem cell transplantation of AML patients. J Clin Med (2020) 9(1):39. doi: 10.3390/jcm9010039 PMC7019914 31878060

[63] Foley B Cooley S Verneris MR Curtsinger J Luo X Waller EK . NK cell education after allogeneic transplantation: dissociation between recovery of cytokine-producing and cytotoxic functions. Blood (2011) 118(10):2784–92. doi: 10.1182/blood-2011-04-347070 PMC3172795 21757615

[64] Kennedy PR Felices M Miller JS . Challenges to the broad application of allogeneic natural killer cell immunotherapy of cancer. Stem Cell Res Ther (2022) 13:165. doi: 10.1186/s13287-022-02769-4 35414042 PMC9006579

[65] Morvan M Lanier L . NK cells and cancer: you can teach innate cells new tricks. Nat Rev Cancer (2016) 16:7–19. doi: 10.1038/nrc.2015.5 26694935

[66] Devillier R Calmels B Guia S Taha M Fauriat C Mfarrej B . Phase I trial of propphylactic donor-derived il-2-Activated nk cell infusion after allogeneic hematopoietic stem cell transplantation from a matched sibling donor. Cancers (Basel) (2021) 13(11):2673. doi: 10.3390/cancers13112673 34071607 PMC8198961

[67] Shapiro RM Birch GC Hu G Vergara Cadavid J Nikiforow S Baginska J . Expansion, persistence, and efficacy of donor memory-like NK cells infused for posttransplant relapse. J Clin Invest (2022) 132(11):e154334. doi: 10.1172/JCI154334 35349491 PMC9151697

[68] Ciurea SO Kongtim P Soebbing D Trikha P Behbehani G Rondon G . Decrease post-transplant relapse using donor-derived expanded NK-cells. Leukemia (2022) 36:155–64. doi: 10.1038/s41375-021-01349-4 PMC8727305 34312462

[69] Jaiswal RS Bhakuni P Bhagawati G Malini Aiyer H Soni M Sharma N . CTLA4Ig-primed donor lymphocyte infusions following haploidentical transplantation improve outcome with a distinct pattern of early immune reconstitution as compared to conventional donor lymphocyte infusions in advanced hematological malignancies. Bone Marrow Transplant (2021) 56:185–94. doi: 10.1038/s41409-020-01002-1 32704091

[70] Chen Y Fukuda T Thakar MS Kornblit BT Storer BE Santos EB . Immunomodulatory effects induced by cytotoxic T lymphocyte antigen 4 immunoglobulin with donor peripheral blood mononuclear cell infusion in canine major histocompatibility complex-haplo-identical non-myeloablative hematopoietic cell transplantation. Cytotherapy. (2011) 13:1269–80. doi: 10.3109/14653249.2011.586997 PMC3195840 21846291

[71] Roy DC Walker I Maertens J Lewalle P Olavarria E Selleslag D . ATIR101 administered after T-cell-Depleted haploidentical HSCT reduces NRM and improves overall survival in acute leukemia. Leukemia (2020) 34:1907–23. doi: 10.1038/s41375-020-0733-0 PMC7326707 32047237

[72] Maung KK Chen BJ Barak I Li Z Rizzieri DA Gasparetto C . Phase I dose escalation study of naive T-cell depleted donor lymphocyte infusion following allogeneic stem cell transplantation. Bone Marrow Transplant (2021) 56:137–43. doi: 10.1038/s41409-020-0991-5 PMC10805111 32624583

[73] Ho VT Kim HT Kao G Cutler C Levine J Rosenblatt J . Sequential infusion of donor-derived dendritic cells with donor lymphocyte infusion for relapsed hematologic cancers after allogeneic hematopoietic stem cell transplantation. Am J Hematol (2014) 89:1092–6. doi: 10.1002/ajh.23825 25132538

[74] Schmidt-Wolf IG Negrin RS Kiem HP Blume KG Weissman IL . Use of a SCID mouse/human lymphoma model to evaluate cytokine-induced killer cells with potent antitumor cell activity. J Exp Med (1991) 174:139–49. doi: 10.1084/jem.174.1.139 PMC2118875 1711560

[75] Verneris MR Kornacker M Mailänder V Negrin RS . Resistance of ex vivo expanded CD3+CD56+ T cells to fas-mediated apoptosis. Cancer Immunol Immunother (2000) 49:335–45. doi: 10.1007/s002620000111 PMC11037019 10946816

[76] Merker M Salzmann-Manrique E Katzki V Huenecke S Bremm M Bakhtiar S . Clearance of hematologic malignancies by allogeneic cytokine-induced killer cell or donor lymphocyte infusions. Biol Blood Marrow Transplant (2019) 25:1281–92. doi: 10.1016/j.bbmt.2019.03.004 30878607

[77] Introna M Lussana F Algarotti A Gotti E Valgardsdottir R Micò C . Phase II study of sequential infusion of donor lymphocyte infusion and cytokine-induced killer cells for patients relapsed after allogeneic hematopoietic stem cell transplantation. Biol Blood Marrow Transplant (2017) 23:2070–8. doi: 10.1016/j.bbmt.2017.07.005 28712935

[78] Rotiroti MC Buracchi C Arcangeli S Galimberti S Valsecchi MG Perriello VM . Targeting CD33 in chemoresistant AML patient-derived xenografts by CAR-CIK cells modified with an improved SB transposon system. Mol Ther (2020) 28(9):1974–86. doi: 10.1016/j.ymthe.2020.05.021 PMC7474266 32526203

[79] Nie Y Yang G Song Y Zhao X So C Liao J . DNA Hypermethylation is a mechanism for loss of expression of the HLA class I genes in human esophageal squamous cell carcinomas. Carcinogenesis (2001) 22(10):1615–23. doi: 10.1093/carcin/22.10.1615 11577000

[80] Atanackovic D Luetkens T Kloth B Fuchs G Cao Y Hildebrandt Y . Cancer-testis antigen expression and its epigenetic modulation in acute myeloid leukemia. Am J Hematol (2011) 86:918–22. doi: 10.1002/ajh.22141 21898529

[81] Orskov AD Treppendahl MB Skovbo A Holm MS Friis LS Hokland M . Hypomethylation and up-regulation of PD-1 in T cells by azacytidine in MDS/AML patients: A rationale for combined targeting of PD-1 and DNA methylation. Oncotarget (2015) 6(11):9612–26. doi: 10.18632/oncotarget.3324 PMC4496243 25823822

[82] Santourlidis S Trompeter HI Weinhold S Eisermann B Meyer KL Wernet P . Crucial role of DNA methylation in determination of clonally distributed killer cell ig-like receptor expression patterns in NK cells. J Immunol (2002) 169:4253–61. doi: 10.4049/jimmunol.169.8.4253 12370356

[83] Choi J Ritchey J Prior JL Holt M Shannon WD Deych E . *In vivo* administration of hypomethylating agents mitigate graft-versus-host disease without sacrificing graft-versus-leukemia. Blood (2010) 116:129–39. doi: 10.1182/blood-2009-12-257253 PMC2904576 20424188

[84] Goodyear OC Dennis M Jilani NY Loke J Siddique S Ryan G . Azacitidine augments expansion of regulatory T cells after allogeneic stem cell transplantation in patients with acute myeloid leukemia (AML). Blood (2012) 119:3361–9. doi: 10.1182/blood-2011-09-377044 22234690

[85] Jabbour E Giralt S Kantarjian H Garcia-Manero G Jagasia M Kebriaei P . Low-dose azacitidine after allogeneic stem cell transplantation for acute leukemia. Cancer (2009) 115(9):1899–905. doi: 10.1002/cncr.24198 PMC4086213 19235255

[86] de Lima M Giralt S Thall PF de Padua Silva L Jones RB Komanduri K . Maintenance therapy with low-dose azacitidine after allogeneic hematopoietic stem cell transplantation for recurrent acute myelogenous leukemia or myelodysplastic syndromes. Cancer (2010) 116(23):5420–31. doi: 10.1002/cncr.25500 PMC5669059 20672358

[87] Pusic I Choi J Fiala MA Gao F Holt M Cashen AF . Maintenance therapy with decitabine after allogeneic stem cell transplantation for acute myelogenous leukemia and myelodysplastic syndrome. Biol Blood Marrow Transplant (2015) 21:1761–9. doi: 10.1016/j.bbmt.2015.05.026 PMC4568135 26055299

[88] Platzbecker U Wermke M Radke J Oelschlaegel U Seltmann F Kiani A . Azacitidine for treatment of imminent relapse in MDS or AML patients after allogeneic HSCT: results of the RELAZA trial. Leukemia (2012) 26(3):381–9. doi: 10.1038/leu.2011.234 PMC3306138 21886171

[89] Platzbecker U Middeke JM Sockel K Herbst R Wolf D Baldus CD . Measurable residual disease-guided treatment with azacitidine to prevent haematological relapse in patients with myelodysplastic syndrome and acute myeloid leukaemia (RELAZA2): an open-label, multicentre, phase 2 trial. Lancet Oncol (2018) 19(12):1668–79. doi: 10.1016/S1470-2045(18)30580-1 30442503

[90] Craddock C Jilani N Siddique S Yap C Khan J Nagra S . Tolerability and clinical activity of post-transplantation azacitidine in patients allografted for acute myeloid leukemia treated on the RICAZA trial. Biol Blood Marrow Transplant (2016) 22(2):385–90. doi: 10.1016/j.bbmt.2015.09.004 PMC4728172 26363443

[91] Vij R Le-Rademacher J Laumann K Hars V Owzar K Shorer T . A phase II multicenter study of the addition of azacitidine to reduced-intensity conditioning allogeneic transplant for high-risk myelodysplasia (and older patients with acute myeloid leukemia): Results of CALGB 100801 (Alliance). Biol Blood Marrow Transplant (2019) 25(10):1984–92. doi: 10.1016/j.bbmt.2019.06.007 PMC6790289 31212080

[92] Oran B de Lima M Garcia-Manero G Thall PF Lin R Popat U . A phase 3 randomized study of 5-azacitidine maintenance vs. observation after Transplant high-risk AML MDS patients Blood Adv (2020) 4:5580–8. 10.1182/bloodadvances.2020002544 PMC7656915 33170934

[93] Gao L Zhang Y Wang S Kong P Su Y Hu J . Effect of rhG-CSF combined with decitabine prophylaxis on relapse of patients with high-risk MRD-negative AML after HSCT: An open-label, multicenter, randomized controlled trial. J Clin Oncol (2020) 38:4249–59. doi: 10.1200/JCO.19.03277 PMC7768335 33108244

[94] Garcia-Manero G Gore SD Kambhampati S . Efficacy and safety of extended dosing schedules of CC-486 (oral azacitidine) in patients with lower-risk myelodysplastic syndromes. Leukemia. (2016) 30:889–96. doi: 10.1038/leu.2015.265 PMC4832070 26442612

[95] de Lima M Oran B Champlin R Papadopoulos EB Giralt S Scott BL . CC-486 maintenance after stem cell transplantation in patients with acute myeloid leukemia or myelodysplastic syndromes. Biol Blood Marrow Transplant (2018) 24(10):2017–24. doi: 10.1016/j.bbmt.2018.06.016 PMC8059405 29933073

[96] Oran B Alatrash G Alousi AM Mehta RS Hosing C Popat UR . Maintenance treatment with guadecitabine (SGI-110) in high risk MDS and AML patients after allogeneic stem cell transplantation. Blood (2020) 136(S1):29–30. doi: 10.1182/blood-2020-134272

[97] Lagadinou ED Sach A Callahan K Rossi RM Neering SJ Minhajuddin M . BCL-2 inhibition targets oxidative phosphorylation and selectively eradicates quiescent human leukemia stem cells. Cell Stem Cell (2013) 12:329–41. doi: 10.1016/j.stem.2012.12.013 PMC3595363 23333149

[98] Zhao P Ni M Ma D Fang Q Zhang Y Li Y . Venetoclax plus azacitidine and donor lymphocyte infusion in treating acute myeloid leukemia patients who relapse after allogeneic hematopoietic stem cell transplantation. Ann Hematol (2022) 101(1):119–30. doi: 10.1007/s00277-021-04674-x PMC8720738 34568973

[99] Amit O On YB Perez G Shargian-Alon L Yeshurun M Ram R . Venetoclax and donor lymphocyte infusion for early relapsed acute myeloid leukemia after allogeneic hematopoietic cell transplantation. a retrospective multicenter trial. Ann Hematol (2021) 100(3):817–24. doi: 10.1007/s00277-021-04398-y 33442793

[100] Schuler E Wagner-Drouet EM Ajib S Bug G Crysandt M Dressler S . Treatment of myeloid malignancies relapsing after allogeneic hematopoietic stem cell transplantation with venetoclax and hypomethylating agents-a retrospective multicenter analysis on behalf of the German cooperative transplant study group. Ann Hematol (2021) 100(4):959–68. doi: 10.1007/s00277-020-04321-x PMC8448702 33191481

[101] Sockel K Bornhauser M Mischak-Weissinger E Trenschel R Wermke M Unzicker C . Lenalidomide maintenance after allogeneic HSCT seems to trigger acute graft-versus-host disease in patients with high-risk myelodysplastic syndromes or acute myeloid leukemia and del(5q): results of the LENMAINT trial. Haematologica (2012) 97(9):e34. doi: 10.3324/haematol.2012.067629 22952334 PMC3436225

[102] Tajima F Adachi K Mochida H Kawatani T Suzumiya J . Effect of azacitidine on CD4+C25+Foxp3+ regulatory T cell on immunity in myelodysplastic syndrome survivors. Blood (2017) 130(S1):1703. doi: 10.1182/blood.V130.Suppl_1.1703.1703

[103] Craddock C Slade D De Santo C Wheat R Ferguson P Hodgkinson A . Combination lenalidomide and azacitidine: A novel salvage therapy in patients who relapse after allogeneic stem-cell transplantation for acute myeloid leukemia. J Clin Oncol (2019) 37:580–8. doi: 10.1200/JCO.18.00889 PMC6494237 30653424

[104] Schroeder T Stelljes M Christopeit M Schmidt E Scheid C Mikesch JH . Treatment of MDS, AML and CMML relapse after allogeneic blood stem cell transplantation with azacitidine, lenalidomide and donor lymphocyte infusions - final results of the prospective azalena-trial (NCT02472691). Blood (2021) 138(S1):411. doi: 10.1182/blood-2021-153282

[105] Kiyoi H Kawashima N Ishikawa Y . FLT3 mutations in acute myeloid leukemia: therapeutic paradigm beyond inhibitor development. Cancer Sci (2020) 111(2):312–22. doi: 10.1111/cas.14274 PMC7004512 31821677

[106] Metzelder SK Schroeder T Lübbert M Ditschkowski M Götze K Scholl S . Long-term survival of sorafenib-treated FLT3-ITD-positive acute myeloid leukaemia patients relapsing after allogeneic stem cell transplantation. Eur J Cancer (2017) 86:233–9. doi: 10.1016/j.ejca.2017.09.016 29055209

[107] Burchert A Bug G Fritz LV Finke J Stelljes M Röllig C . Sorafenib maintenance after allogeneic hematopoietic stem cell transplantation for acute myeloid leukemia with *FLT3*-internal tandem duplication mutation (SORMAIN). J Clin Oncol (2020) 38(26):2993–3002. doi: 10.1200/JCO.19.03345 32673171

[108] Brunner AM Li S Fathi AT Wadleigh M Ho VT Collier K . Haematopoietic cell transplantation with and without sorafenib maintenance for patients with FLT3-ITD acute myeloid leukaemia in first complete remission. Br J Haematol (2016) 175(3):496–504. doi: 10.1111/bjh.14260 27434660 PMC5083189

[109] Tschan-Plessl A Halter JP Heim D Medinger M Passweg JR Gerull S . Synergistic effect of sorafenib and cGvHD in patients with high-risk FLT3-ITD+AML allows long-term disease control after allogeneic transplantation. Ann Hematol (2015) 94(11):1899–905. doi: 10.1007/s00277-015-2461-5 26233683

[110] Pratz KW Levis M . How I treat FLT3-mutated AML. Blood. (2017) 129(5):565–71. doi: 10.1182/blood-2016-09-693648 PMC5290983 27872057

[111] Xuan L Wang Y Huang F Fan Z Xu Y Sun J . Sorafenib maintenance in patients with FLT3-ITD acute myeloid leukaemia undergoing allogeneic haematopoietic stem-cell transplantation: an open-label, multicentre, randomised phase 3 trial. Lancet Oncol (2020) 21(9):1201–12. doi: 10.1016/S1470-2045(20)30455-1 32791048

[112] Mathew NR Baumgartner F Braun L O'Sullivan D Thomas S Waterhouse M . Sorafenib promotes graft-versus-leukemia activity in mice and humans through IL-15 production in FLT3-ITD-mutant leukemia cells. Nat Med (2018) 24:282–91. doi: 10.1038/nm.4484 PMC6029618 29431743

[113] Bazarbachi A Bug G Baron F Brissot E Ciceri F Dalle IA . Clinical practice recommendation on hematopoietic stem cell transplantation for acute myeloid leukemia patients with FLT3-internal tandem duplication: a position statement from the acute leukemia working party of the European society for blood and marrow transplantation. Haematologica. (2020) 105(6):1507–16. doi: 10.3324/haematol.2019.243410 PMC7271578 32241850

[114] Rautenberg C Nachtkamp K Dienst A Schmidt PV Heyn C Konakci M . Sorafenib and azacitidine as salvage therapy for relapse of FLT3-ITD mutated AML after allo-SCT. Eur J Hematol (2017) 98(4):348–54. doi: 10.1111/ejh.12832 27893163

[115] Schlenk RF Weber D Fiedler W Salih HR Wulf G Salwender H . German-Austrian AML study group. midostaurin added to chemotherapy and continued single-agent maintenance therapy in acute myeloid leukemia with *FLT3*-ITD. Blood (2019) 133(8):840–51. doi: 10.1182/blood-2018-08-869453 30563875

[116] Maziarz RT Levis M Patnaik MM Scott BL Mohan SR Deol A . Midostaurin after allogeneic stem cell transplant in patients with FLT3-internal tandem duplication-positive acute myeloid leukemia. Bone Marrow Transplant (2021) 56(5):1180–9. doi: 10.1038/s41409-020-01153-1 PMC8113057 33288862

[117] Levis M Perl AE . Gilteritinib: potent targeting of FLT3 mutations in AML. Blood Adv (2020) 4(6):1178–91. doi: 10.1182/bloodadvances.2019000174 PMC7094008 32208491

[118] Perl AE Martinelli G Cortes JE Neubauer A Berman E Paolini S . Gilteritinib or chemotherapy for relapsed or refractory *FLT3*-mutated AML. N Engl J Med (2019) 381(18):1728–40. doi: 10.1056/NEJMoa1902688 31665578

[119] Brunner MC Chambers CA Chan FK Hanke J Winoto A Allison JP . CTLA-4-Mediated inhibition of early events of T cell proliferation. J Immunol (1999) 162(10):5813–20. doi: 10.4049/jimmunol.162.10.5813 10229815

[120] Engelhardt JJ Sullivan TJ Allison JP . CTLA-4 overexpression inhibits T cell responses through a CD28-B7-Dependent mechanism. J Immunol (2006) 177(2):1052–61. doi: 10.4049/jimmunol.177.2.1052 16818761

[121] Korman AJ Garrett-Thomson SC Lonberg N . The foundatios of immune checkpoint blockade and the ipilimumab approval decennial. Nat Rev Drug Discovery (2022) 21(7):509–28. doi: 10.1038/s41573-021-00345-8 34937915

[122] Graf M Reif S Hecht K Pelka-Fleischer R Kroell T Pfister K . High expression of costimulatory molecules correlates with low relapse-free survival probability in acute myeloid leukemia (AML). Ann Hematol (2005) 84(5):287–97. doi: 10.1007/s00277-004-0978-0 15592672

[123] Davids MS Kim HT Bachireddy P Costello C Liguori R Savell A . Ipilimumab for patients with relapse after allogeneic transplantation. N Engl J Med (2016) 375(2):143–53. doi: 10.1056/NEJMoa1601202 PMC5149459 27410923

[124] Garcia JS Flamand Y Tomlinson BK Keng M Mendez LM Khaled S . Safety and efficacy of decitabine plus ipilimumab in relapsed or refractory MDS/AML in the post-BMT or transplant naïve settings. Blood (2020) 136(S1):15–7. doi: 10.1182/blood-2020-136235

[125] Montalban-Bravo G DiNardo CD . The role of IDH mutations in acute myeloid leukemia. Future Oncol (2018) 14(10):979–93. doi: 10.2217/fon-2017-0523 29543066

[126] Salhotra A Afkhami M Yang D Mokhtari S Telatar M Gu D . Allogeneic hematopoietic cell transplantation outcomes in patients carrying isocitrate dehydrogenase mutations. Clin Lymphoma Myel Leuk (2019) 19(7)e400–5. doi: 10.1016/j.clml.2019.04.007 PMC9129101 31155409

[127] Konopleva M Martinelli G Andreeff Daver N Papayannidis C Wei A . MDM2 inhibition: an important step forward in cancer therapy. Leukemia (2020) 34:2858–74. doi: 10.1038/s41375-020-0949-z 32651541

[128] Ho JNHG Schmidt D Lowinus T Ryoo J Dopfer EP Nunez NG . Targeting MDM2 enhances antileukemia immunity after allogeneic transplantation *via* MHC-II and TRAIL-R1/2 upregulation. Blood (2022) 140(10):1167–81. doi: 10.1182/blood.2022016082 PMC9461473 35853161

[129] Wolf Y Anderson AC Kuchroo VK . TIM3 comes of age as an inhibitory receptor. Nat Rev Immunol (2020) 20:173–85. doi: 10.1038/s41577-019-0224-6 PMC7327798 31676858

[130] Brunner AM Traer E Vey N . Allogeneic hematopoietic cell transplantation outcomes of patients with R/R AML of higher-risk MDS treated with the TIM-3 inhibitor MBG453 (Sabatolimab) and hypomethylating agents. Blood (2021) 138 (Supplement 1):3677. doi: 10.1182/blood-2021-151455

